# Ascites and Enterocolitis in a Preterm Infant with Acquired CMV Infection: A Case Study and Review of the Literature

**DOI:** 10.3390/jcm14165854

**Published:** 2025-08-19

**Authors:** Keren Nathan, Ellen Bamberger, Daniel Dubin, Morya Shneider, Narmin Shehade Smair, Rasha Zoabi Safadi

**Affiliations:** 1 Department of Pediatric Infectious Disease, Bnai Zion Medical Center, Haifa 3339419, Israel; 2 Technion Israel Institute of Technology, Rappaport, Haifa 3525422, Israelnarmeen.smair@gmail.com (N.S.S.);; 3 Department of Pediatric Surgery, Bnai Zion Medical Center, Haifa 3339419, Israel; 4 Department of Neonatology, Bnai Zion Medical Center, Haifa 3339419, Israel; 5 Department of Pediatric Gastroenterology, Bnai Zion Medical Center, Haifa 3339419, Israel

**Keywords:** postnatal cytomegalovirus (CMV), very low birth weight (VLBW), ascites, gastrointestinal complications

## Abstract

Postnatal cytomegalovirus (pCMV) infection is typically asymptomatic in term infants but poses significant risks to very preterm and very low birth weight (VLBW) infants. The primary mode of transmission of pCMV is breast milk from seropositive mothers. Here, we present the case of a 29-week preterm female who contracted pCMV and began to manifest symptoms at day of life (DOL) 50. She developed respiratory compromise, massive ascites, and was extremely ill. The patient was managed with ganciclovir (GCV), intravenous immunoglobulins (IVIG), and percutaneous drainage of the ascites. She gradually improved and was discharged after a 5-month neonatal intensive care unit (NICU) stay. After presenting the case, we review the clinical manifestations of pCMV, and particularly its less well-recognized gastrointestinal manifestations, including ascites. We then outline guidelines for treatment and prevention. Clinicians should consider pCMV in VLBW and extremely premature infants presenting with thrombocytopenia, colitis, or ascites, especially in the second and third months of life.

## 1. Introduction

Postnatal CMV (pCMV) infection is mostly asymptomatic in term infants, but can cause significant morbidity and mortality in preterm infants. Very preterm infants, with gestational age (GA) <32 weeks and very low birth weight (VLBW) <1500 g, are an especially vulnerable population [1]. Maternal milk from a seropositive mother is the main mode for transmission of pCMV infection in such infants. Diagnosis of pCMV can be made after the age of 21 days based on the detection of CMV DNA from urine, blood, and CSF or respiratory secretions, with the exclusion of congenital CMV (cCMV). Diagnosis of cCMV is made before the age of 21 days by DNA PCR of CMV from urine, blood, or CSF. After 21 days, the diagnosis can be made retrospectively by PCR on dried blood spots (DBS). DBS analysis has a sensitivity of 85% and specificity of 100% [2,3]. Therefore, a negative PCR on DBS does not exclude the diagnosis of cCMV, whereas a positive test is diagnostic for cCMV.

We present a case of a preterm infant with pCMV infection manifesting gastrointestinal symptoms and, most interestingly, severe ascites. Ascites is an uncommon presentation in preterm infants and is exceedingly rare in pCMV. Following that, we review gastrointestinal manifestations, including ascites secondary to pCMV.

## 2. Case Presentation

This is the case of a 29 + 0-week female infant, the first of monochorionic diamniotic twins born after placental abruption. Selective intra-uterine growth restriction (IUGR) in this twin (Twin #1) was noted early in the pregnancy. The IUGR was thought to be due to placental insufficiency, with reversed placental diastolic flow; there were no signs of twin-to-twin transfusion. Birth weight was 740 g, below the 10th percentile (Fenton growth charts); the other twin (Twin #2) had a birth weight in the 40th percentile. While the infant was vigorous at birth with Apgar scores of 7 and 8 at 1 and 5 min, respectively, she required non-invasive respiratory support. Her infectious workup was negative, including a negative CMV PCR saliva, which is routinely performed on all neonates in our institution on day of life (DOL) #1. Maternal CMV serology was consistent with a past infection. On DOL #3, the infant developed abdominal distention and was diagnosed with spontaneous intestinal perforation (SIP). A percutaneous drain was placed, and wide-spectrum antibiotic therapy was initiated. Despite conservative treatment, gastrointestinal symptoms persisted, and on DOL #17, she underwent a laparotomy with ileostomy placement. She gradually improved and was placed on enteral feeding consisting alternately of cow’s milk-based formula and her own mother’s milk due to insufficient own-mother milk volumes. The patient’s stool was non-diarrheal (ileostomy), and she was gaining adequate weight. From a respiratory standpoint, she required a high-flow nasal cannula and was considered to have evolving bronchopulmonary dysplasia (BPD).

On DOL #50, the infant developed respiratory distress with increasing oxygen requirement, apneas, poor perfusion with cutis marmorata, and loose bloody stools. Notably, her abdomen was non-distended and soft, and she was tolerating feeds. Laboratory tests revealed leukopenia (WBC 3370 cells/µL) and neutropenia (absolute neutrophil count 900/µL) with mild thrombocytopenia, and mildly elevated CRP. Electrolytes, creatinine, and liver enzymes were within the normal range. Blood gas revealed mild respiratory acidosis with non-elevated lactate. Chest and abdominal X-ray showed non-specific findings with evolving chronic lung disease and bowel loop distension. At this point, the differential diagnosis included localized gastrointestinal infection, systemic infectious etiology, FPIES (food protein-induced enterocolitis syndrome), or late surgical complications.

**Figure 1:**
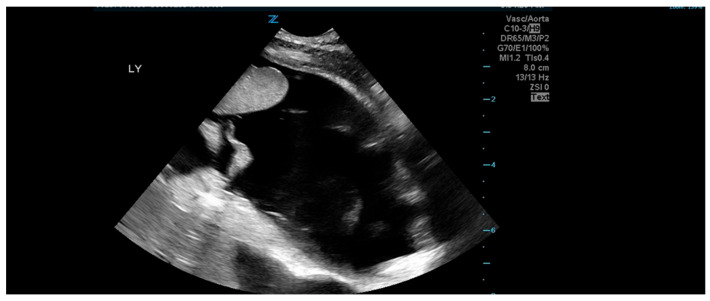
Sonographic imaging of the ascites.

**Figure 2:**
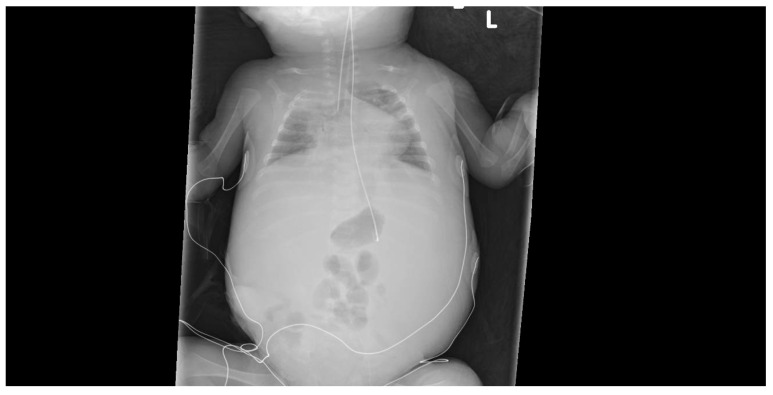
Ascites and reduced lung volume.

Microbiological testing revealed normal blood, urine, and stool cultures. Additionally, a nasopharyngeal PCR was negative for (respiratory) viral pathogens, and an echocardiogram showed no signs of pulmonary hypertension. The infant’s cow’s milk-based formula was changed to a hypoallergenic hydrolyzed formula. Last, her urine PCR for CMV DNA was repeated and has now tested positive. Accordingly, pCMV was diagnosed. Notably, her twin sister (Twin #2) also tested positive for urine CMV PCR but remained asymptomatic. Twin #1 was treated with IV ganciclovir (GCV), a 6 mg/kg dose twice a day. Six days into the treatment, on DOL #60, her condition deteriorated. Specifically, she developed significant abdominal distention and respiratory distress, necessitating intubation and mechanical ventilation. Abdominal sonography (Figure 1) showed massive ascites with normal liver size and texture. Her respiratory distress was attributed to the elevated intra-abdominal pressure related to the massive ascites (Figure 2). Albumin level was 2.6 mg/dL, and liver function tests were normal (e.g., liver enzymes and coagulation profile were within normal limits).

As there was no suggestion of liver dysfunction, the ascites was attributed to third spacing (i.e., significant capillary leaking) associated with systemic inflammatory response syndrome caused by the CMV infection. An alternative explanation was protein-losing enteropathy due to CMV colitis. The ascitic fluid was removed by percutaneous drainage and demonstrated transudative features. Bacterial and fungal cultures from the fluid were negative. Unfortunately, CMV PCR was not tested at this stage. However, given the mechanism that we suggest for the ascites, a negative result would not have excluded the diagnosis or altered our management.

**Figure 3:**
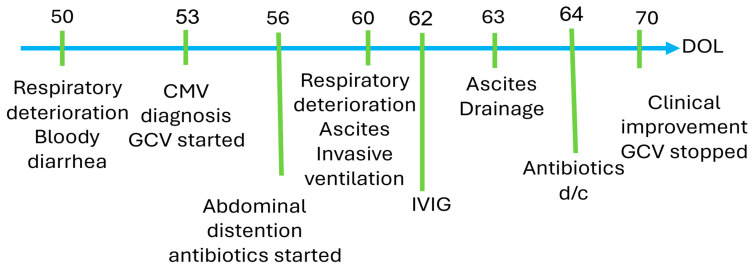
Patient’s clinical events.

**Figure 4:**
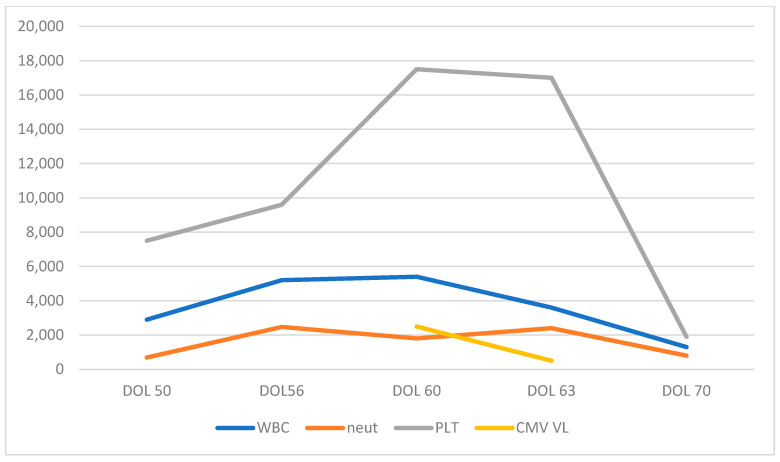
Cell blood count and CMV viral load over time. WBC—white cell count cells/µL, neut—neutrophil cells/µL, and PLT—platelets cells/µL × 10.

Due to her worsening clinical status under GCV treatment, the infant was given a single dose of IVIG. She began to improve clinically on DOL# 64. CMV viral load in the blood 7 days after the beginning of treatment was 2800 copies, and it continued to decrease after two weeks of treatment. GCV was discontinued after three weeks of treatment because of severe neutropenia, which resolved within a week. Gradual improvement continued, with significant respiratory improvement allowing extubation on DOL #72, and complete resolution of abdominal ascites by DOL #83 Figure 3 and Figure 4.

The infant was discharged from the NICU at the age of 130 days with a diagnosis of severe BPD and received treatment with inhaled corticosteroid therapy and complete oral feeding on high-calorie formula. A cranial ultrasound performed at discharge was normal, with no calcifications or other abnormalities. The infant also passed a hearing screen in both ears.

## 3. Clinical Presentation of Gastrointestinal Involvement in pCMV—Review of the Literature

### 3.1. Epidemiology

Postnatal CMV in infants is acquired mostly from the breast milk (BM) of seropositive mothers. Over 96% of seropositive mothers will reactivate and excrete CMV into the milk at some point after delivery [4]. The reactivation is local to the mammary glands, without dissemination or a localized infectious focus in the mother. The virions are found in BM cells and cell-free whey [5]. Not all infants become infected when consuming CMV-positive milk: the reported rate of infection via breast milk in exposed babies ranges between 5.7% and 58.6% [6]. In addition, the majority of infants who acquire pCMV remain asymptomatic, with only 30% developing symptoms. The peak of CMV DNA appearance in breast milk is 4–6 weeks postpartum [7]. The major risk factor for acquiring pCMV is low gestational age, and the risk increases as gestational age decreases. CMV viral load in breast milk consumed by infected infants is also significantly higher than in milk given to those who are not infected.

Although pCMV can lead to significant morbidity, studies suggest that it does not impact mortality [8,9]. The mortality rate reported by Kelly et al. was 1.2% [10].

### 3.2. Pathophysiology

While cCMV is transmitted to the fetus transplacentally following maternal viremia, pCMV is transmitted from BM via three possible routes: (1) the trans-olfactory route, where the virus enters through the nose and travels along the olfactory nerve; (2) the trans-mucosal route, where the virus enters via the mouth or nose during direct feeding; and (3) the trans-gastric route, where the virus enters the small intestine via intestinal epithelial cells. The latter is the main route of transmission in preterm infants fed by gastric or jejunal tubes [11]. The virus replicates in the transmission site and then continues to replicate in the lymph nodes, and eventually in the reticuloendothelial system [12].

Another possible mode of CMV transmission is via blood products. However, leukoreduced or seronegative blood transfusions administered to neonates at our institution (and many others) make this possibility highly unlikely, as such processing greatly reduces transmission [13,14]

Gestational age is also an important factor in the acquisition of CMV. Preterm infants are more prone to infection due to an immature immune system, reduced transplacental transfer of maternal anti-CMV IgG antibodies, and underdeveloped gastrointestinal mucosa. As noted above, not all exposed infants are infected. Infection is probably influenced by other comorbidities associated with prematurity, such as gastrointestinal insults or severe BPD [15,16].

### 3.3. Clinical Manifestations

Symptomatic pCMV in preterm infants is usually diagnosed in the second or third months of life. The clinical picture is non-specific and may resemble many other infectious causes. Moreover, non-infectious conditions such as BPD, necrotizing enterocolitis (NEC), and others can be mistaken for pCMV. Indeed, the clinical manifestations of pCMV are diverse, affecting multiple organ systems, and vary widely in severity. Below, we review the common manifestations of pCMV, with particular focus on gastrointestinal involvement.

#### 3.3.1. Non-Gastrointestinal Manifestations

**Sepsis-like syndrome** (SLS), mimicking bacterial sepsis with apneas, bradycardia, and gray pallor, is estimated to appear in up to 4.5% of VLBW infants with pCMV in the U.S. [17]. Much higher rates, up to 30–60%, have also been reported in retrospective studies which included extremely preterm and VLBW infants [8,18]. **Marrow suppression with thrombocytopenia** and, to a lesser extent neutropenia, are frequently found. In a large multicenter retrospective study by Kelly et al. with 303 patients diagnosed with pCMV, 66% presented with thrombocytopenia and 34% with neutropenia [10]. Minihan et al. suggested that thrombocytopenia could be an early marker for pCMV since it can precede the diagnosis by 2–3 weeks [8]. This finding can be the sole manifestation and may serve as an indication for CMV testing in preterm infants. **Pneumonitis** can be present as part of multiorgan involvement in SLS or may occur alone. The clinical manifestations of CMV pneumonitis include diffuse interstitial inflammation. Pulmonary involvement can progress to fibrosis and BPD with a reported risk ratio of 1.33 and a risk ratio of 1.2 for death at 36 weeks of gestation, as demonstrated in the largest cohort to date, which identified 328 affected infants out of 101,111 that were screened in a multicenter study [10]. **Hepatitis** and hepatomegaly with hyperbilirubinemia and elevated liver enzymes were also reported in some studies [6,8,19]. Short-term outcomes of pCMV were reported in a retrospective study of 48 pCMV preterm infants compared to 48 matched preterm control infants. The study found that days of ventilation and length of stay in days were significantly higher for affected infants compared to non-affected (23.5 vs. 12 and 140 vs. 110.5 days, respectively) [8]. The impact of pCMV on **neurodevelopmental impairment** (NDI) has been evaluated in two retrospective cohort studies, each including over 50 infants assessed at 2 years of age. Both studies found no significant difference in NDI between infants with and without pCMV [8,20]. However, in a large systematic review that included 40 studies with 1975 cases, conflicting evidence was found regarding the long-term outcome in infants with pCMV. Interpretation of these results is challenging due to confounding factors common in preterm infants, such as complications of prematurity causing global developmental delay or socioeconomic factors. Some studies with shorter follow-up periods (6 months to 4.5 years) found no difference in NDI, whereas studies with longer follow-ups (3–17 years) noted a small reduction in IQ scores among preterm infants with pCMV, although the number of cases was small (19 infants) [21]. Interestingly, while cCMV is the most common non-genetic cause of hearing loss in infants, which can present at birth or progressively in the first two years of life, infants with pCMV are not at increased risk for hearing loss, as was shown over 2268 cases [22].

#### 3.3.2. Gastrointestinal Manifestations

Gastrointestinal (GI) involvement is not thought to be a prominent feature of pCMV. While bloody diarrhea often prompts CMV testing, infants with other GI conditions, such as **necrotizing enterocolitis**, are not commonly investigated for CMV. However, NEC, with and without perforations, and subsequent complications such as strictures and volvulus have been reported in association with pCMV, in a study of 47 cases of pCMV with GI involvement [23]. The authors of that study, Goelz et al., suggested that intestinal involvement could be more common than previously thought. Association with NEC was also demonstrated in 11/16 cases of pCMV with GI involvement in a single center [24].

Necrotizing enterocolitis affects approximately 7% of preterm infants [25]. Many studies have shown that human breast milk has a protective effect against NEC [26], and yet in the case of pCMV infection, BM is the putative cause. The role of CMV in the development of NEC remains difficult to estimate, as data on the prevalence of CMV in NEC or SIP is limited and inconsistent. In one retrospective study analyzing 70 intestinal specimens from 61 infants with NEC or SIP, CMV was detected in 81% [27], but in another study, it was only found in 4% of 178 specimens over a 16-year period [28]. Interestingly, a large meta-analysis of 24 studies found that viral infections such as rotavirus, norovirus, astrovirus, and CMV were significantly associated with NEC, but causality could not be determined [29]. Furthermore, Patel et al. [30] showed that the prevalence of NEC in infants <1500 g was much higher in those with pCMV (6/33, 18%) compared to infants not infected with CMV (37/563, 6.6%). They found an adjusted cause-specific HR of 2.81, which shows an association between NEC and pCMV but is not statistically significant [30]. The typical clinical presentation of NEC and pCMV includes abdominal distention, dilated bowel loops, and bloody stools with or without evidence of pneumonitis intestinalis on abdominal X-rays [31,32,33,34]. Atypical presentations of NEC have been reported in association with evidence of CMV infection, such as ileal perforation [35], or as a late presentation of abdominal compartment syndrome, dilated loop, and bloody diarrhea in a 27-week gestational-age infant presented on DOL #74. Additionally, in reviewing pathologic specimens from nine additional cases of CMV enterocolitis, Lee et al. concluded that CMV enterocolitis can be distinguished from NEC by the presence of ulceration and strictures rather than gangrene and perforation [36]. Thrombocytopenia was present in all cases, and management included surgery and antibiotic treatment. In some cases, the diagnosis was made through pathological examination of resected tissue showing evidence of CMV. In addition, one case report had evidence of CMV DNAemia several weeks before the onset of sepsis-like syndrome and CMV-associated NEC [31]. As noted by the authors of that study, these findings underscore the importance of including CMV in the differential diagnosis of sepsis and NEC, and also may suggest preemptive monitoring. All cases of CMV-associated NEC, except for one baby [32], were treated with GCV and had a good overall prognosis. Therefore, it is important to consider CMV testing in any case of NEC with atypical features or without the typical radiological signs of NEC [24].

**Colitis with bloody diarrhea** can be an isolated manifestation of pCMV or precede CMV-associated NEC and other GI or non-GI manifestations of CMV, such as pneumonitis and sepsis-like syndrome. Bloody diarrhea can be present in cases of CMV colitis, as illustrated in the case of a preterm VLBW infant born at 24 weeks of GA who developed symptoms on DOL #60. The infant received a four-week course of GCV. At 18 months of age, the infant exhibited mild developmental delay and no evidence of hearing loss [37]. Severe colitis, while rare, can be the presenting symptom in term infants. One report involved a 2-month-old baby who presented with bloody diarrhea and colonic stenosis [38]. In another series, 10 term babies with a median age of 2.5 months presented with intractable diarrhea and ulcerations of the colon and rectum. Some received GCV treatment, but all had clinical improvement and a favorable prognosis [39]. Another series of three term infants presented at 2–3 months with intractable or hemorrhagic diarrhea with CMV detected in stool. They were treated with GCV and oral valGCV [40].

**Intussusception** is extremely rare in preterm infants; moreover, the diagnosis is often delayed due to the clinical overlap with NEC, which is far more common. Accurate diagnosis is important since management strategies for these conditions differ. CMV was implicated as a trigger for intussusception in a preterm infant born at 24 weeks of gestation, who was diagnosed with pCMV infection on DOL #62 and later developed ileo–ileal intussusception on DOL #92. The infant was managed with surgical resection and GCV therapy [41].

**Ascites** is a rare presentation in preterm infants, and its incidence is not well established. Cases of fetal ascites are typically monitored postnatally. The etiology in this population includes urinary tract abnormalities, gastrointestinal perforation, congenital infections, cardiac failure, chylous ascites, and metabolic or genetic disorders [42]. Ascites secondary to cCMV infection has been previously described, even as a sole presentation without hepatitis. Proposed mechanisms include hypoalbuminemia, portal hypertension, and hepatic vein obstruction resulting from cCMV [43]. To the best of our knowledge, our case of massive ascites in a preterm infant with pCMV is only the second case reported since 1989. The previously described case involved a 5-week-old term infant with mild hepatitis, thrombocytopenia, and two ascites episodes eight weeks apart. Diagnosis was based on a fourfold rise in the CMV IgG titers and positive urine CMV. The authors suggested that portal hypertension secondary to hepatitis was the cause of ascites [44]. Reports of fetal ascites related to cCMV are scant. In one case, ascites was diagnosed in a fetus at 28 weeks of gestation, with maternal blood positive in a CMV PCR. At birth, the newborn exhibited other cCMV-related symptoms and complications, including IUGR, microcephaly, hepatitis, and thrombocytopenia, and it was later found to have bilateral sensorineural hearing loss. The infant was treated with GCV for six weeks, and the ascites resolved by eight weeks of age [45]. In two other case reports of fetal CMV, massive ascites causing pulmonary hypoplasia is described. Despite fetal interventions including treatment with immunoglobulins and in utero ascites drainage, both infants died shortly after birth [46].

### 3.4. Treatment

Currently, there are no consensus guidelines or randomized control trials addressing the treatment of symptomatic pCMV. Most available data on the efficacy and safety of GCV treatment pertain to symptomatic cCMV in term infants. Both GCV and oral valGCV have been shown to suppress viral replication and are associated with improved neurodevelopmental outcomes and modest hearing improvement at one year of age in cCMV [47,48]. In pCMV, several cohort studies did not demonstrate a difference in clinical outcome between infants treated with GCV and those who were not. For example, a case–control study of 172 infants born before 32 weeks of gestation found no significant differences in the incidence of chronic lung disease, length of stay (LOS), or death in the 20% that received antiviral therapy compared with those who did not [9]. Similarly, other studies with smaller cohorts of treated infants found no significant difference in short-term outcomes such as BPD, death, or LOS, and in cognitive impairment at two years [8,20]. Data on the pharmacokinetics of GCV in VLBW are limited. A case report demonstrated that therapeutic drug monitoring of GCV in a VLBW infant led to a marked suppression of viral load and clinical improvement when the dosing interval was adjusted based on plasma drug levels [49]. IVIG has also been used as a treatment option, though less commonly. In a cohort of 24 infants where 17 received IVIG, and 7 of those were treated with both IVIG and GCV, no long-term benefit was observed with GCV therapy (the benefit of dual therapy was not assessed) [18].

Treatment with GCV for two to three weeks with surgical resection for NEC has been shown to improve GI symptoms as well as other pCMV manifestations such as thrombocytopenia and hepatocellular involvement [31]. In a review by Bar-Meir et al. [34], 11 out of 28 patients received GCV. Resolution of GI symptoms was observed, with supportive therapy and surgical resections in case of obstruction. Similarly, pCMV enterocolitis cases treated with surgery and GCV also demonstrated complete resolution of symptoms [36].

### 3.5. Prevention

BM provides significant benefits to VLBW infants, including lower incidence of NEC, late-onset sepsis, chronic lung disease, retinopathy of prematurity, and neurodevelopmental impairment [50]. Strategies to prevent CMV transmission through BM focus on pasteurization and freezing, and also monitoring for CMV in BM. The evidence regarding the elimination of the virus from BM is mixed. Omarsdottier et al. [51] showed that freezing fresh BM does not eliminate the virus completely in VLBW and extreme preterm infants. Transmission occurred in 3/37 (8%) in the BM freezing group vs. 2/33 (6%) in the untreated BM [51]. On the other hand, another report noted that freeze–thawing BM resulted in elimination of the virus in around 85% of samples and reduced viral load in the rest [52].

The two main methods for pasteurization are Holder pasteurization (62 degrees for 30 min), which is the gold standard, and short-term pasteurization (62 degrees for 5 s). Taking both together, pasteurization showed promising results in the largest systematic review and meta-analysis [53], with a reduction of 82% of pCMV infections in VLBW infants. Transmission occurred at rates of 13% in untreated BM, 6.1% in frozen BM, and 2.3% in pasteurized BM. Short-term pasteurization showed promising results in one study, with reduced CMV transmission of 2.3% (2/87) compared with transmission of 20% in untreated BM (17/83)—a risk ratio of 8.2 [54].

Macronutrients are stable under all treatment methods, but bioactive elements are diminished by Holder pasteurization [53]. However, treating BM by either freezing or pasteurization can have an influence on the content of other components in the milk, as shown during the preparation of donor BM. Both freezing and pasteurization, along with container changing and prolonged storage, influence the bioactivity of BM, so that it preserves the ability to protect from NEC but not necessarily against other complications such as late-onset sepsis, chronic lung disease, retinopathy of prematurity, and neurodevelopmental impairment [50]. Currently, freezing is not advised. Although it may reduce viral load, it does not change the risk of SLS and is thought to reduce the bioavailability of mother’s milk [52]. That said, there are no formal guidelines regarding BM treatment, and variability between institutions exists. Other promising methods, such as microwave irradiation, may demonstrate some benefits in the future.

At our institution, all full-term healthy newborn babies undergo universal screening for cCMV by pooled saliva PCR testing [55]. In our NICU, maternal BM is not routinely screened for CMV, nor is it subjected to freezing or pasteurization. Fresh BM is given without any special processing regardless of maternal seropositivity. In the present case, we observed definite evidence of CMV in both twins, with clear confirmation of postnatal CMV. Notably, one twin who was growth-restricted developed complicated and symptomatic pCMV, while the other, although also infected with CMV, was completely asymptomatic and had a favorable course. This observation, especially the fact that twin #2 had no prior gastrointestinal insults and was gaining weight, suggests that host factors and comorbidities strongly influence the clinical presentation of pCMV. Since there was no other obvious cause, and abdominal distention, colitis, and thrombocytopenia were present, we decided to treat with GCV in the first instance. Later, the development of massive ascites further exacerbated respiratory distress and pneumonitis. When the clinical response was not satisfactory, we added IVIG on the basis of other studies that included this treatment [18]. Combined management with antiviral GCV, IVIG, and therapeutic aspiration of ascites led to clinical improvement and resolution.

As noted above, to our knowledge, this is the first reported case of pCMV-associated severe ascites secondary to symptomatic pCMV infection since 1989. We hypothesize that the ascites resulted from significant protein loss due to bloody colitis and systemic capillary leak syndrome in a preterm infant with a prior history of spontaneous intestinal perforation. It has been discussed previously that a defect in the intestinal wall, as in SIP, could predispose patients to communicating intraluminal intestinal content with ascites [56]. We acknowledge that there is a gap in our case of about 40 days between the insult and the clinical presentation of the ascites, but the contribution of the defect should be considered.

Gastrointestinal manifestations of postnatal CMV appear in the literature. However, overall, the level of evidence varies and is mostly limited to case series and case reports. Specifically, NEC is described more in association with CMV in clinical cases and was found in the pathology specimen after resection. Other manifestations are scantly described, and ascites, on the other hand, is rarely described. Still, one must recognize the importance of the association between postnatal CMV and GI pathologies, and the need to raise awareness among healthcare providers managing VLBW and extremely premature patients. 

## 4. Conclusions

Intestinal manifestations of pCMV are likely underdiagnosed and may be more common than previously recognized. VLBW and extreme premature infants who receive BM feeding from seropositive mothers and develop thrombocytopenia during the second and third months of life should be evaluated for pCMV. Moreover, this diagnosis should also be considered in infants presenting with colitis and or ascites and even atypical NEC. Indeed, while ascites is a rare occurrence in pCMV, timely recognition and possible initiation of GCV therapy should be considered. Given the established benefits of fresh BM and the low incidence of symptomatic and severe pCMV infection, we do not recommend any change in breast milk preparation. However, clinicians should maintain a low threshold for considering this evolving diagnosis in at-risk infants.

## Abbreviations

The following abbreviations are used in this manuscript:

VLBW	very low birth weight
GCV	ganciclovir
NEC	necrotizing enterocolitis
BPD	bronchopulmonary dysplasia
LOS	length of stay
IVIG	intravenous immunoglobulins
NDI	neurodevelopmental impairment
CMV	cytomegalovirus
pCMV	postnatal CMV
GI	gastrointestinal
cCMV	congenital CMV
NICU	neonatal intensive care unit
SIP	spontaneous intestinal perforation
